# “Current dementia care: what are the difficulties and how can we advance care globally?”

**DOI:** 10.1186/s12913-020-05307-1

**Published:** 2020-05-13

**Authors:** Clarissa Giebel

**Affiliations:** 1 National Institute of Health Research Applied Research Collaboration North West Coast (NIHR ARC NWC), Liverpool, UK; 2 Institute of Population Health Sciences, University of Liverpool, Liverpool, UK

## Abstract

**Background:**

Dementia is a growing global public health concern, with post-diagnostic care often very limited. Depending on where people live, both within a country and depending on high-, middle-, and low-income countries, they might also face barriers in accessing the right care at the right time. Therefore, it is important to highlight recent evidence on the facilitators and barriers to dementia care, but also evidence on how to move dementia care forward.

**Main text:**

Current dementia care is subject to several inequalities, including living in rural regions and being from a minority ethnic background. Evidence in this collection highlights the issues that both people living with dementia and unpaid carers are facing in accessing the right care, with evidence from Australia, Canada, Uganda, to the Netherlands, and further afield. Providing improved dementia-specific training to health care professionals and supporting medication and reablement interventions have been identified as possible ways to improve dementia care for all.

**Conclusions:**

This special issue focuses on recent evidence on inequalities in dementia care across the globe and how dementia care can be advanced in various areas.

*BMC Health Services Research* is pleased to launch ‘*Advancing Dementia Care’*, an article collection focused on current dementia care inequalities and what can be done to advance care. Dementia affects an estimated 50 million people worldwide [1], with numbers steadily growing. This can affect many areas in a person’s life – from struggling to do the shopping and managing medication [2] to behavioral difficulties and cognitive problems [3]. Many people with dementia have unpaid carers (family members or friends) [4] who help them with daily tasks ranging from preparing a meal to more personal tasks such as washing and dressing. However, carers are most often overlooked when it comes to supporting those people affected by dementia.

To support both people with dementia and their carers, adequate post-diagnostic support needs to be in place (which can include memory groups, support groups, respite care, day-care centers, and social activities in the community). Not everyone receives the same level of support, for multiple reasons. The World Health Organisation (WHO) defines health inequities as “differences in health status or in the distribution of health resources between different population groups, arising from the social conditions in which people are born, grow, live, work and age. Health inequities are unfair and could be reduced by the right mix of government policies.” Looking at the barriers of accessing post-diagnostic care across eight European countries, for example, Broda and colleagues [5] reported a lack of awareness and continuity of care as some of the main issues from the perspectives of policy and political decision-makers. However, some carers reject accessing specific types of post-diagnostic support, such as support groups, particularly when the person they care for is experiencing higher levels of everyday functioning and thus in less need of support [6].

One underlying reason for inequalities, that receives growing attention, is rural dementia care. Living in more rural communities can make it difficult to access both a GP or memory clinic for a diagnosis, but also to access support services afterwards [7]. If it takes an hour to access a support group, which only meets once a week, due to reduced demand, then people living in more rural regions will be unable to access the same level of post-diagnostic support than those living in more urban regions. Looking at the barriers and facilitators of implementing a strategy for dementia assessment and management in rural Canada, Morgan and colleagues [8] found that whilst it proved difficult to implement such a strategy in primary health care, the intervention proved to be successful also due to the strong partnership between researchers and clinicians. But this is just one example of how rural dementia care is being tackled.

Belonging to an ethnic minority group can also be leading to inequalities in diagnosis and care access in dementia [9]. People from black and minority ethnic (BAME) groups are often found to experience delays in receiving a diagnosis, with symptoms also being attributed to faith by some resulting in delays or general non-contact with GPs about their symptoms [10]. In line with other research on various BAME groups, Sagbaken et al. [11] reported language barriers and faith-related help-seeking attitudes as major barriers to receiving a dementia diagnosis in ethnic minority migrants in Norway. Similarly, GPs were reported to often lack cultural competency, which further created barriers to a timely diagnosis. A recent scoping review by Bieber and colleagues [12] further supports these findings, as ethnicity was found to be a barrier to service access in dementia. But ethnic minority status also leads to inequalities in accessing post-diagnostic care, including anti-dementia medication [13].

Living in rural areas or being from a BAME background are only some of the barriers to receiving the right post-diagnostic care and diagnosis in the first place. A growing body of evidence is showing that being from more disadvantaged backgrounds, for example, is linked to reduced access to anti-dementia medication [14], with more research currently being conducted on how neighborhood deprivation (measured via the Index of Multiple Deprivation, in the UK) is linked to dementia care.

Dementia care can differ not only within a country and by postcode, but also between countries. Whilst the Netherlands are renowned for their advanced dementia care both in the community and in long-term care, there are huge variations between high-income and low-and middle-income countries (LMIC). Kamoga et al. [15] explored dementia diagnosis and treatment in Uganda and found that health care workers generally had very little training on recognizing and treating dementia symptoms. Those health care workers who were able to recognize symptoms were more likely also to focus on treating other medical symptoms as opposed to those related to dementia. This suggests that there is a need for improved dementia training in the health care workforce, and possibly a better knowledge exchange across countries.

Barriers to care are not only reported in the community though, but also in long-term care institutions and thus the more advanced stages of the condition. By conducting focus groups with nursing home staff in Norway, Midtbust and colleagues [16] reported various barriers in providing palliative care in dementia. These included the lack of time spent with individual residents, as well as the frequently temporary nature of staffing. These staffing issues are not only reflective of problems in palliative care, however, but also generally in providing adequate care in long-term care institutions.

While there are many barriers and inequalities to receiving the right dementia care at the right time, there are several ways in which dementia care can be advanced though. First and foremost, getting a timely diagnosis is key, which means that barriers to getting a diagnosis, by for example people from BAME groups, need to be removed. In their paper, Watson and colleagues [17] reported how 92% of a sample of Australian health service consumers preferred a dementia diagnosis as soon as possible. This does help indeed to provide suitable care (including medication and post-diagnostic services) in time to reduce the speed of progression. Providing better support in re-enabling people with dementia is also vital and forms part of the post-diagnostic care (which also includes medication management [18]. In the Australian aged care sector, O’Connor and colleagues [19] showed that reablement interventions for people with dementia are varied, and to improve access to and usage of interventions, these should be multi-faceted with a freely available resource outlining the different intervention components. This is supported to a degree by a recent meta-analysis by Backhouse et al. [20], showing mixed evidence on the benefits of coordinated community interventions. Tackling daily functioning does not always need to involve the actual person living with dementia however, but medication management, for example, can also be addressed via community pharmacy-based interventions [18].

To advance dementia care, we need to take a global view and learn from research and clinical practice across the globe. Findings from Australia might provide important insights for improving dementia care in rural Canada or LMICs for example, and barriers identified in the Netherlands might also be relevant to the UK. This article collection thus highlights some of the recent evidence in this field and brings together inequalities research and how inequalities and general dementia care might be tackled in the future.

## Data Availability

Not applicable.
